# Prevalence of sacroiliitis on magnetic resonance enterography in Crohn’s disease and its association with intestinal findings: a monocentric observational cross-sectional study

**DOI:** 10.1007/s10238-025-01846-1

**Published:** 2025-09-04

**Authors:** G. Amati, G. Sandri, A. Bertani, D. Vaccari, A. Pecchi, B. Bongiovanni, M. Orlandi, G. Ciancio, M. Pecchini, O. Secchi, A. Colecchia, P. Torricelli, D. Giuggioli

**Affiliations:** 1 Rheumatology Unit, University Hospital of Modena, Modena, Italy; 2 Chair of Rheumatology, University Hospital of Modena, University of Modena and Reggio Emilia, Policlinico Di Modena, Via del Pozzo, 71, 41125 Modena, Italy; 3 Gastroenterology Unit, University Hospital of Modena, Modena, Italy; 4 Radiology Unit, Hospital of Pavullo, Local Health Unit of Modena, Pavullo, MO Italy; 5 Chair of Radiology, University Hospital of Modena, University of Modena and Reggio Emilia, Modena, Italy; 6 Chair of Gastroenterology, University Hospital of Modena, University of Modena and Reggio Emilia, Modena, Italy

**Keywords:** Sacroiliitis, Axial spondyloarthritis, Crohn’s disease, Inflammatory bowel disease, Magnetic resonance enterography

## Abstract

Magnetic resonance enterography (MRE) is recommended for the assessment of small intestine alterations in Crohn’s disease (CD). Sacroiliac joints (SIJs) imaging has a central role in the early diagnosis of sacroiliitis (SI). MRE can evaluate both acute and structural findings of SIJs. We aimed to assess the prevalence of SI detected by MRE in a cohort of CD patients, and the associations of SI with demographic and clinical features and with intestinal MRE findings. Two hundred patients affected by CD (M:F 1:1, median age 49.5 (22.5) years, median CD duration 4.75 (16.2) years) tested with MRE between 2011 and 2023 were selected. They discontinued tumor necrosis factor α inhibitors (TNFαi) at least 3 months before the MRE execution. Most patients had an ileal CD location (65.0%) and a stricturing behavior of disease (50.0%). Thirty-five percent of patients underwent ileocecal resection. One out of ten patients were treated with at least one TNFαi. Active SI, capsulitis, erosions, sclerosis, and ankylosis were present in 10.5%, 0.5%, 2.0%, 2.5%, and 1.5%, respectively. No significant correlations have been evidenced between the presence of SI and demographic and clinical variables. The presence of an asymmetric hyperenhancement of the bowel wall was instead directly associated with the presence of SI (OR 8.61, 95% CI 1.47–50.4, *p* = 0.017). In this study, subclinical SI is a frequent finding in CD patients being present in one out of ten MRE examination. This phenomenon was significantly associated with asymmetric mural enhancement, a specific CD intestinal lesion at MRE.

## Introduction

Inflammatory bowel diseases (IBDs) are chronic inflammatory diseases with unknown etiology, including ulcerative colitis (UC) and Crohn’s disease (CD) [1]. Due to the systemic behavior of the disease, about half of IBD patients are affected by various extraintestinal manifestations (EIMs) such as ocular (uveitis, episcleritis), cutaneous (erythema nodosum, pyoderma gangrenosum), hepatobiliary (sclerosing cholangitis), and joint disease [2]. IBD-arthritis is one of the most frequent EIMs among IBD patients and is classified under the group of spondyloarthritis (SpA), a spectrum of chronic inflammatory musculoskeletal diseases which can present both axial (sacroiliitis (SI), spondylitis) and peripheral (arthritis, tenosynovitis, peritendinitis, enthesitis) involvement [2]. Both axial and peripheral IBD-arthritis—the latter known as “enteropathic arthritis” (EA)—can occur in CD; however, axial involvement seems to be more frequent in CD than in UC [3–5]. According to the Assessment of the Spondyloarthritis Society (ASAS) classification criteria, axial SpA (axSpA) includes radiographic axSpA—or ankylosing spondylitis—and non-radiographic axSpA [3, 4]. The diagnosis of a radiographic axSpA requires the evidence of overt articular damage of sacroiliac joints (SIJs) assessed by means of conventional X-rays according to the modified New York diagnostic criteria [4, 6, 7]. Non-radiographic axSpA relies on a combination of clinical history, physical examination, laboratory data, and axial magnetic resonance imaging (MRI) and does not require radiographic evidence of SI [4, 8]. Based on available epidemiological data, spondylitis and SI occur in about 2–16% and 6–46% of IBD patients, respectively, while the prevalence of EA ranges from 17–62% [9]. Considering that involvement of SIJs occurs earlier and is more severe compared to spinal involvement, imaging of SIJs has a central role in the early diagnosis of axSpA [10]. Since the publication of the ASAS classification criteria in 2009, MRI has become the gold standard for the assessment of SIJs thanks to its capacity to detect active SI in the early stages of the disease in the form of bone marrow edema (BME) by means of STIR sequences [4, 6, 7]. The criteria of SI highly suggestive of axSpA were published in the same year by the ASAS MRI study group and then periodically updated and validated [8, 11, 12]. To date, SIJs MRI is the method of choice for detecting active inflammatory changes in the early stages of the disease as well as highlighting chronic structural signs with precise anatomical localization [8, 11, 12]. Magnetic resonance enterography (MRE) is widely used to assess both inflammatory activity and structural damage of CD [13]. MRE imaging entails a variety of axial and coronal T1- and T2-weighted sequences of the small bowel. An evaluation of the potentially affected skeletal component is also possible including the SIJs in coronal and axial views. These scans can therefore be used to assess the SIJs for SI features including joint erosions, ankylosis, and juxta-articular signal changes. However, the radiological assessment of the aforementioned aspects is often overlooked and exceeds the initial request for MRE [14, 15]. Cross-sectional imaging of the bowel has been used in the past to study the prevalence of subclinical SI in IBD patients, varying from 2 to 68% for computed tomography [15].

The aim of the present study is to investigate the prevalence of SI detected by MRE in a cohort of CD patients who underwent MRE examination for gastroenterological purposes and the association of SI with demographic data, clinical features, immunosuppressive therapies, with a focus on intestinal findings during the same MRE examination.

## Methods

### Population and study design

This is a monocentric observational cross-sectional study performed in CD patients followed by the IBD outpatient clinic of the University Hospital of Modena, a tertiary referral center for IBDs equipped with a joint gastro-rheumatological outpatient clinic. The inclusion criteria were: a diagnosis of CD [16], the presence of at least one MRE testing between January 1, 2011 and December 31, 2023, and the discontinuation of tumor necrosis factor α inhibitors (TNFαi) for at least 3 months prior to MRE. The latter criterion was chosen to avoid the potential influence of TNFαi treatment on SIJs inflammation.

The exclusion criteria were the presence of other diagnosed rheumatic diseases, the diagnosis of SpA prior to MRE, the unavailability of a full scan of SIJs, and the absence of the main variables of interest.

The research project was approved by the local Institutional Ethics Committee (Comitato Etico Area Vasta Emilia Nord, protocol no. 7461/25), and written informed consent was obtained from all participants.

### Data collection

Data collection was managed with an electronic case report form. Data on demographic and clinical variables were obtained from all CD patients and included sex, age, and CD duration at MRE execution, CD features according to Montreal classification [17], personal or family history of psoriasis (PsO), family history of SpA and IBD, smoking habit, HLA-B27 status, previous ileocecal resection, ongoing mesalazine treatment, and previous immunosuppressant treatments (at least six months of uninterrupted therapy). Apart from the rheumatological assessment in suspected (and subsequently confirmed) cases of IBD-arthritis, no additional data were available due to the retrospective nature of the study.

### Assessment of the MRE evaluation

The study coordinator (GA) assigned a unique numerical code to each MRE examination and presented all the MREs to one reader (DV). Ambiguous cases were evaluated by a radiologist with over 20 years’ experience in body MRI (AP). All the readers were blinded to the patients' clinical data and assessed the presence of SI in each MRE examination using a dedicated workstation (Synapse, Fujifilm Medical).

### Imaging technique

All MRI scans were performed using high-field (1.5 T) scanners: (1) Achieva (Philips Healthcare); (2) Ingenia (Philips Healthcare); (3) Signa Voyager (General Electric Healthcare). We adopted the standard protocol for MRE used in our Centre, which included the following imaging sequences: (1) T1w standard, axial; (2) bSSFP (BTFE/FIESTA), axial and coronal; (3) T2w standard, single-shot, axial and coronal; (4) T2w fat-sat, single-shot, axial, with fat suppression achieved with the SPAIR (spectral-attenuated inversion recovery) technique; (5) DWI (diffusion weighted imaging) with increasing b values (0, 500, 1000) and resulting ADC (apparent diffusion coefficient) mapping; (6) Unenhanced T1w fat-sat (THRIVE/LAVA), with fat suppression achieved with the SPAIR technique, axial and/or coronal; (7) Enhanced T1w fat-sat (THRIVE/LAVA), with fat suppression achieved with the SPAIR technique, axial and/or coronal, using multiple enhanced scans. For the enhanced scans, we used the standard paramagnetic contrast medium, Gd-DOTA or gadoteric acid (Dotarem, Guerbet) at the usual dosing (0.2 ml/kg). Intestinal distention was achieved by administering, at least 30–60 min prior to scanning, 1.5 L of oral hyperosmotic solution (Macrogol 4000).

### Articular and intestinal lesion definitions

**Fig. 1:**
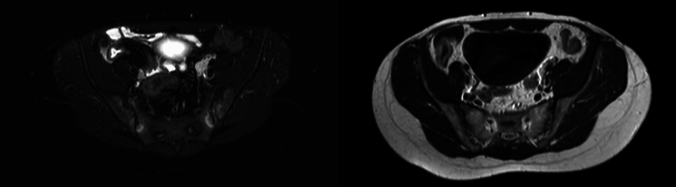
Axial MRI scans of the sacroiliac joints show bilateral sacroiliitis, with subchondral bone marrow edema appearing as hyperintense signal on T2-weighted SPAIR images (left) and corresponding hypointense signal on T1-weighted images (right), consistent with active sacroiliitis according to the 2019 ASAS MRI criteria [12]

MRE was considered positive for SI when active inflammatory changes involving the SIJs were present according to the ASAS MRI study group’s most updated definition [11, 12]. In particular, active SI was defined as a hyperintense signal at the subchondral bone on T2-weighted sequence sensitive for free water (e.g., short tau inversion recovery, spectral-attenuated inversion recovery, or T2-weighted fat suppressed) in at least two consecutive slices or a single slice, provided there was more than one inflammatory lesion present (Fig. 1). Concerning other inflammatory lesions and chronic structural changes, readers evaluated capsulitis, enthesitis, joint space fluid, fat metaplasia, erosions, sclerosis, ankylosis, and non-bridging bone buds.

The intestinal involvement of CD patients on MRE was assessed according to the most updated recommendations of the Crohn’s Disease focused panel of the Society of Abdominal Radiology [18]. The following parameters were collected: presence of segmental mural enhancement and its pattern (asymmetric, stratified, homogeneous), wall thickening measured in millimeters, intramural edema, stenosis, ulcers, perienteric inflammatory edema, signs of diminished motility, presence of engorged vasa recta, fibrofatty proliferation, mesenteric venous thrombosis and/or occlusion, regional lymphadenopathy, occurrence of fistulas, inflammatory abscess or mass, and/or perforation. An active CD was defined for the presence of at least one inflammatory lesion on MRE.

### Statistical analysis

Categorical variables are described as frequencies, whereas quantitative variables are reported as median and interquartile range (IQR). Comparison between groups was carried out with binomial logistic regression. Statistical significance was defined as a p-value lower than or equal to 0.05. Statistical analyses were performed with Jamovi statistical software (version 2.3.21.0).

## Results

### Selection of the population

In the period between January 1, 2011 and December 31, 2023, 610 MREs were performed out of 4262 magnetic resonance examinations requested by the Gastroenterology Unit, involving a total of 388 patients. All patients without a clear CD diagnosis were excluded, corresponding to 471 MREs and 272 patients. All MREs performed without a full SIJs visualization were excluded (415 MREs and 255 patients remained). Six patients were subsequently excluded due to lack of clinical information, three patients were excluded for other rheumatic diagnosis, and finally nine patients were excluded for SpA diagnosis prior to CD diagnosis, leaving 237 patients.

All patients with an ongoing treatment with TNFαi or those who discontinued the treatment less than 3 months prior to MRE were excluded, resulting in a final cohort of 200 patients.

### Description of the population

A total of 200 CD patients were selected. Fifty-three percent (21/200) of patients were of male sex and the median age was of 49.5 (IQR 22.5) years. The median CD duration was of 4.75 (IQR 16.2) years and the median age of CD diagnosis was 32.2 (IQR 21.7) years. Sixty-three percent (126/200) of patients were diagnosed between the age of 17 and 40, according to Montreal classification. One-hundred thirty patients (65.0%) had an ileal CD location and 21 out of 200 had a concomitant upper involvement. Half of the sample had a stricturing behavior of the disease. A concurrent perianal involvement was present in 16.0% (32/200) of patients and the 35.0% (70/200) of the sample underwent ileocecal resection. Seventy-three percent of patients (146/200) were on treatment with mesalazine, and one out of ten was treated with at least one TNFα inhibitor. Detailed demographical and clinical data are reported in Table 1.

**Table 1:** descriptive statistics of demographic and clinical variables, and ongoing therapies

	Missing	Total (*n* = 200)
Male sex (n, %)	0	107 (53.5)
Median age (IQR)	0	45.9 (22.5)
Median CD duration (IQR)	0	4.75 (16.2)
Age at CD diagnosis (IQR)	0	32.2 (21.7)
Montreal classification		
*Age at CD diagnosis*	0	
A1—less than 16 years (n, %)		8 (4.0)
A2—between 17 and 40 years (n, %)		126 (63.0)
A3—higher than 40 years (n, %)		66 (33.0)
*Disease location*	0	
L1—ileal (n, %)		130 (65.0)
L2—colonic (n, %)		14 (7.0)
L3—ileo-colonic (n, %)		56 (28.0)
L4—upper disease (n, %)		21 (28.0)
*Disease behaviour*	0	
B1—non-stricturing/non-penetrating (n, %)		80 (40.0)
B2—stricturing (n, %)		104 (52.0)
B3—penetrating (n, %)		44 (22.0)
B2 + B3—stricturing and penetrating (n, %)		28 (14.0)
Perianal disease (n, %)	0	32 (16.0)
Ileocecal resection (n, %)	0	70 (35.0)
PsO (n, %)	172	18 (64.3)
Family history of PsO (n, %)	178	10 (45.5)
Family history of IBD (n, %)	120	18 (22.5)
Family history of SpA (n, %)	185	6 (40.0)
HLA-B27 (n, %)	182	3 (16.7)
Smoking habit		
Active (n, %)	90	54 (49.1)
Previous (n, %)	90	26 (23.6)
Never (n, %)	90	30 (27.3)
Mesalazine (ongoing) (n, %)	0	146 (73.0)
Azathioprine (past or ongoing) (n, %)	8	8 (4.2)
Azathioprine duration (Median, IQR)		2.38 (4.44)
Sulfasalazine (past or ongoing) (n, %)	3	0 (0.0)
At least one TNFαi (n, %)		19 (9.6)
Adalimumab (past) (n, %)	1	14 (7.0)
Adalimumab duration (Median, IQR)		3.71 (2.37)
Infliximab (past) (n, %)	2	6 (3.0)
Infliximab duration (Median, IQR)		1.96 (2.29)
Vedolizumab (past or ongoing) (n, %)	0	11 (5.5)
Vedolizumab duration (Median, IQR)		1.53 (1.90)

*CD* Crohn’s disease, *IBD* inflammatory bowel disease, *IQR* interquartile range, *PsO* psoriasis, *SpA* spondyloarthritis, *TNFα* tumor necrosis factor α inhibitor

### Frequencies of evaluated MRE lesions

According to the definition provided by the ASAS/MRI working group, 10.5% of CD patients presented SI, 14.3% of which were bilateral. Most of the sacroiliac inflammatory involvement was only on the iliac side (75.0%), while 20.8% of SI had both iliac and sacral inflammatory involvement. Only one patient displayed capsulitis (0.5%) and 2.0% of patients presented erosions. Sclerosis and ankylosis were found in 2.5% and 1.5% of patients, respectively. All patients presenting ankylosis had a bilateral involvement. None presented enthesitis, joint space fluid, or non-bridging bone buds.

More than half of patients had an active CD according to the presence of inflammatory bowel lesions (69.5%), while 8% of patients had a complicated disease (Table 2).

**Table 2:** frequencies of MRE lesions found in the sample

	Missing	Total (*n* = 200)
Overall sacroiliitis (n, %)	0	21 (10.5)
Bilateral sacroiliitis (n, % overall)		3 (14.3)
Monolateral sacroiliitis (n, % overall)		18 (85.7)
Only iliac involvement (n, % involved sacroiliac joints)		18 (75.0)
Only sacral involvement (n, % involved sacroiliac joints)		1 (4.2)
Iliac and ischiatic involvement (n, % involved sacroiliac joints)		5 (20.8)
Capsulitis (n, %)	0	1 (0.5)
Entesitis (n, %)	0	0 (0.0)
Joint space fluid (n, %)	0	0 (0.0)
Erosions (n, %)	0	4 (2.0)
Fat metaplasia (n, %)	0	0 (0.0)
Overall sclerosis (n, %)	0	5 (2.5)
Bilateral sclerosis (n, % overall)		2 (40.0)
Monolateral sclerosis (n, % overall)		3 (60.0)
Only iliac involvement (n, % involved sacroiliac joints)		5 (71.4)
Only sacral involvement (n, % involved sacroiliac joints)		0 (0.0)
Iliac and ischiatic involvement (n, % involved sacroiliac joints)		2 (28.6)
Overall ankylosis (n, %)	0	3 (1.5)
Bilateral ankylosis (n, % overall)		3 (100.0)
Monolateral ankylosis (n, % overall)		0 (0.0)
Non bridging bone bud (n, %)	0	0 (0.0)
Intestinal disease activity (n, %)	0	139 (69.5)
Segmental mural hyperenhancement (n, %)	0	139 (79.5)
Asymmetric (n, %)		9 (6.5)
Stratified (n, %)		89 (64.0)
Homogeneous (n, %)		41 (29.5)
Wall thickening (n, %)	0	149 (74.5)
Mild, from 3 to 5 mm (n, %)		23 (15.4)
Moderate, from 5 to 10 mm (n, %)		93 (62.4)
Severe, higher than 10 mm (n, %)		33 (22.2)
Intramural edema (n, %)	0	121 (60.5)
Stricture (n, %)	0	50 (25.0)
Ulcerations (n, %)	0	0 (0.0)
Diminished motility (n, %)		59 (29.5)
Perienteric edema/inflammation (n, %)	0	91 (45.5)
Engorged vasa recta (n, %)	0	95 (47.5)
Fibrofatty proliferation (n, %)	0	95 (47.5)
Mesenteric venous thrombosis/occlusion (n, %)	0	0 (0.0)
Adenopathy (n, %)	0	118 (59.0)
Complicated disease (n, %)	0	17 (8.5)
Fistulas (n, %)		14 (7.0)
Phlegmon (n, %)		2 (1.0)
Abscess (n, %)		2 (1.0)
Perforation (n, %)		1 (0.5)

### Associations with the presence of SI at the MRE evaluation

No associations have been evidenced between the presence of SI and all demographic and clinical variables, and previous or ongoing therapies (Table 3).

**Table 3:** Univariate analysis of association between the presence of sacroiliitis and clinical variables

	Missing	Presence of Sacroiliitis (*n* = 21)	Absence of Sacroiliitis (*n* = 179)	*p*-value
Male sex (n, %)	0	9 (42.9)	98 (54.8)	0.305
Median age (IQR)	0	46.2 (27.0)	45.6 (22.1)	0.312
Median CD duration (IQR)	0	6.29 (13.0)	4.33 (16.1)	0.426
Age at CD diagnosis (IQR)	0	33.5 (21.1)	32.1 (21.7)	0.639
Montreal classification				
*Age at CD diagnosis*	0			
A1—less than 16 years (n, %)		1 (4.8)	7 (3.9)	0.851
A2—between 17 and 40 years (n, %)		13 (61.9)	113 (63.1)	0.913
A3—higher than 40 years (n, %)		7 (33.3)	59 (33.0)	0.973
*Disease location*	0			
L1—ileal (n, %)		16 (76.2)	114 (63.7)	0.261
L2—colonic (n, %)		0 (0.0)	14 (7.8)	0.988
L3—ileo-colonic (n, %)		5 (23.8)	51 (28.5)	0.652
L4—upper disease (n, %)		2 (9.5)	19 (10.6)	0.878
*Disease behaviour*	0			
B1—nonstricturing/nonpenetrating (n, %)		9 (57.1)	71 (39.7)	0.778
B2—stricturing (n, %)		10 (47.6)	94 (52.5)	0.671
B3—penetrating (n, %)		5 (23.8)	39 (21.8)	0.833
B2 + B3—stricturing and penetrating (n, %)		3 (14.3)	25 (14.0)	0.968
Perianal disease (n, %)	0	5 (23.8)	27 (15.1)	0.568
Ileocecal resection (n, %)	0	8 (38.1)	62 (34.6)	0.861
PsO (n, %)	172	3 (75.0)	15 (62.5)	0.633
Family history of PsO (n, %)	178	2 (66.7)	8 (42.1)	0.440
Family history of IBD (n, %)	120	1 (16.7)	17 (23.0)	0.724
Family history of SpA (n, %)	185	0 (0.0)	6 (46.2)	0.997
HLA-B27 (n, %)	182	1 (33.3)	2 (13.3)	0.308
Smoking habit				0.294
Active (n, %)	90	6 (54.6)	48 (48.5)	
Previous (n, %)	90	4 (36.4)	22 (22.2)	
Never (n, %)	90	1 (9.1)	29 (29.3)	
Mesalazine (ongoing) (n, %)	0	18 (85.7)	128 (71.5)	0.177
Azathioprine (past or ongoing) (n, %)	8	2 (10.0)	6 (3.5)	0.188
Azathioprine duration (Median, IQR)		4.96 (4.46)	2.39 (2.39)	0.929
Sulfasalazine (past or ongoing) (n, %)	3	0 (0.0)	0 (0.0)	n.a
At least one TNFα inhibitor (n, %)	3	2 (9.5)	18 (10.2)	0.985
Adalimumab (past) (n, %)	1	1 (4.8)	13 (7.3)	0.669
Adalimumab duration (Median, IQR)		3.00 (0.00)	4.00 (2.50)	0.726
Infliximab (past) (n, %)	2	1 (4.8)	5 (2.8)	0.628
Infliximab duration (Median, IQR)		0.59 (0.00)	2.92 (2.00)	0.572
Vedolizumab (past or ongoing) (n, %)	0	1 (4.8)	10 (5.6)	0.876
Vedolizumab duration (Median, IQR)		1.53 (0.00)	1.53 (2.15)	0.768

*CD* Crohn’s disease, *IBD* inflammatory bowel disease, *IQR* interquartile range, *PsO* psoriasis, *n.a.* not applicable, *SpA* spondyloarthritis, *TNFα* tumor necrosis factor α

By comparing groups according to the presence of SI, the asymmetric hyperenhancement of the bowel wall was directly associated with SI (*p* = 0.025) (Table 4). The association was confirmed following normalization by means of binary logistic regression according to sex, age and CD duration, with an odds ratio of 8.61 (95% CI 1.47–50.4, *p* = 0.017) (Table 5).

**Table 4:** univariate analysis of association between the presence of sacroiliitis and intestinal lesions found on MRE evaluation

	Missing	Presence of Sacroiliitis (*n* = 21)	Absence of Sacroiliitis (*n* = 179)	*p*-value
Intestinal disease activity (n, %)	0	15 (71.4)	124 (69.3)	0.839
Segmental mural hyperenhancement (n, %)	0	17 (81.0)	122 (68.2)	0.236
Asymmetric (n, %)		3 (14.3)	6 (3.4)	**0.025**
Stratified (n, %)		10 (47.6)	79 (44.1)	0.339
Homogeneous (n, %)		4 (19.1)	37 (20.7)	0.558
Wall thickening (n, %)	0	17 (80.9)	132 (73.7)	0.476
Mild, from 3 to 5 mm (n, %)		3 (14.3)	20 (11.2)	0.484
Moderate, from 5 to 10 mm (n, %)		10 (47.6)	83 (46.4)	0.575
Severe, higher than 10 mm (n, %)		4 (19.1)	29 (16.2)	0.517
Intramural edema (n, %)	0	13 (61.9)	108 (60.3)	0.889
Stricture (n, %)	0	7 (33.3)	43 (24.0)	0.354
Ulcerations (n, %)	0	0 (0.0)	0 (0.0)	n.a
Diminished motility (n, %)	0	8 (38.1)	51 (28.5)	0.364
Perienteric edema/inflammation (n, %)	0	8 (38.1)	83 (46.4)	0.473
Engorged vasa recta (n, %)	0	10 (4.6)	85 (47.5)	0.991
Fibrofatty proliferation (n, %)	0	12 (57.1)	83 (46.4)	0.352
Mesenteric venous thrombosis/occlusion (n, %)	0	0 (0.0)	0 (0.0)	n.a
Adenopathy (n, %)	0	15 (71.4)	103 (57.5)	0.226
Complicated disease (n, %)	0	0 (0.0)	17 (9.5)	0.992
Fistulas (n, %)		0 (0.0)	14 (7.8)	0.988
Phlegmon (n, %)		0 (0.0)	2 (1.1)	0.990
Abscess (n, %)		0 (0.0)	2 (1.1)	0.990
Perforation (n, %)		0 (0.0)	1 (0.6)	0.993

*n.a* not applicable

**Table 5:** normalization of the association found on univariate analysis by means of binary logistic regression including sex, age and CD duration

	Univariate			Multivariate		
	OR	95%CI	*p*-value	OR	95% CI	*p*-value
Male sex	0.62	0.25–1.54	0.305	0.61	0.24–1.57	0.304
Age	3.25	0.18–57.9	0.422	1.83	0.53–6.25	0.720
CD duration	1.02	0.99–1.06	0.242	1.62	0.80–3.29	0.178
*Segmental mural hyperenhancement*						
Asymmetric–negative	7.13	1.28–39.7	**0.025**	8.61	1.47–50.4	**0.017**
Stratified–negative	1.80	0.54–6.04	0.339	1.82	0.53–6.25	0.342
Homogeneous–negative	1.54	0.36–6.54	0.558	1.74	0.40–7.54	0.462

*CD* Crohn’s disease, *CI* confidence interval, *OR* odds ratio

## Discussion

To our knowledge, this is one of the widest cohorts of CD patients where the presence of SI was investigated by means of MRE. The prevalence of active SI defined according to the ASAS MRI study group criteria amounted to 10.5%. Few studies evaluated the phenomenon by means of MRI or MRE, with an overall prevalence of SI from 10.9 to 39.0% in CD patients [19–28]. In these studies, SI was associated with demographic features such as female sex, greater age, and body mass index, and clinical features including a previous ileocecal resection, a longer disease duration, stricturing disease behavior, and an ileocolic disease localization. Among the aforementioned studies, some included established SpA patients, and SI was associated with back pain, history of dactylitis and higher Bath Ankylosing Spondylitis Functional Index scores [19–28]. Unfortunately, these studies lack homogeneity due to different inclusion and exclusion criteria or unique definitions for determining the presence of SI. Furthermore, SI has never been associated with the presence of the MRE-assessed intestinal lesions. The only two studies which reported the prevalence of SI on MRE in a whole sample of CD patients clearly specifying the SI definition, the frequencies of the phenomenon were of 17.0 and 20.0% [23, 28]. Said frequencies are significantly higher than our findings, however, these studies based the definition of SI either on the presence of active SI according to the ASAS MRI criteria or on the presence of structural lesions alone, such as erosions, potentially overestimating the prevalence of SI. In fact, although erosions are considered structural lesions specific of axSpA, a recent systematic review of the literature has found that almost 10% of patients without axSpA might present erosions detected by MRI [29]. No associations between SI and clinical or demographic features have been found in our study. However, by comparing SI positive and negative groups based on the presence of inflammatory and structural intestinal lesions, an association of SI with the presence of asymmetric mural hyperenhancement was demonstrated for the first time. This finding was confirmed following the normalization of the result by age, sex, and disease duration with an odds ratio of 8.61. This finding could serve as a clue for SpA, prompting the gastroenterologist to test for “SpA red flags,” raise suspicion of clinical SI, and refer the patient to a rheumatologist; in fact, early diagnosis is important not only for improving patient outcomes, but also for enabling personalized medical management.

The significance of bowel wall hyperenhancement has been extensively evaluated in computed tomography enterography, where it has been associated with an increased risk of disease relapse, need for surgery, neutrophilic inflammation, and endoscopic disease activity [30–33]. Although fewer studies have investigated the significance of mural hyperenhancement on MRE, the available evidence similarly links it to higher endoscopic activity and disease recurrence [34, 35]. However, data specifically addressing asymmetric mural hyperenhancement remain limited. In existing literature, this pattern has primarily been established in the differential diagnosis of CD versus other inflammatory, neoplastic, or infectious conditions, where it is considered a relatively specific marker of CD [18, 36].

Although direct evidence on clinical implication of this specific pattern is lacking, its known specificity for CD and its association with markers of more active disease suggest that it may reflect a more aggressive inflammatory phenotype, potentially contributing to extraintestinal manifestations such as sacroiliitis. No significant associations were observed with other intestinal imaging markers on MRE.

This study has several strengths. Firstly, this is the second widest cohort described in literature which evaluated SI by means of MRE in CD patients and the first study evaluating the association of this phenomenon with inflammatory and structural intestinal lesions. Moreover, this study has been conducted in one of the national referral centers for IBDs.

The present study also has some limitations: The first concerns the retrospective design of the study which did not make it possible to collect the data regarding the rheumatic symptoms of the patients enrolled. Moreover, most patients of the sample had never been evaluated by a rheumatologist according to our healthcare software; therefore, the assumption was that said patients had never experienced any articular pain worthy of further investigation.

This assumption inevitably led us to also include patients with an occult inflammatory axial involvement, as demonstrated by the presence of patients with articular damage such as ankylosis (three patients with bilateral ankylosis, 1.5% of the whole sample).

Secondly, the most relevant risk factors of SpA, such as HLA locus B status or personal and/or family history of PsO, were not available for all patients due to the fact that these were not routinely assessed in CD patients by the gastroenterologists.

Moreover, the monocentric nature of the study may not be representative of the global CD population, despite the fact that patients had been enrolled in a third-level center for IBDs.

In its current state, SI does not seem to represent a predictor of SpA in CD patients, in contrast to previous EIMs and arthralgia, which are associated with an increased risk of developing CD-related arthritis [37]. It should be noted that despite the absence of this association, in the cohort described by Giovannini and colleagues, only six cases of peripheral SpA had developed during the follow-up; therefore, the association—or absence of association—with axSpA with SI could not be stated.

Lastly, considering that BME has multiple differential diagnoses, the etiology of the suspected inflammatory lesion should be differentiated according to the localization proposed by some authors [38].

As previously stated by other authors, wider cohorts are needed, by developing complex study protocols including all the potential variables which characterize both CD and SpA phenotypes and the most updated protocols for SIJs and spine evaluation [39, 40].

## Conclusions

In this study, subclinical SI is a frequent finding in CD patients being present in one out of ten MRE examinations. This phenomenon was associated with asymmetric mural enhancement, a specific CD intestinal lesion at MRE. The importance of this result would extend to both radiology and gastroenterology practice, leading the specialist to suspect SI and prompting an early referral of the patient to a rheumatologist for a possible diagnosis of axSpA.

## Data Availability

No datasets were generated or analysed during the current study.

## Abbreviations

ASAS: Assessment of spondyloarthritis society
axSpA: Axial spondyloarthritis
BME: Bone marrow edema
CD: Crohn’s disease
CI: Confidence interval
EIMs: Extraintestinal manifestations
IBDs: Inflammatory bowel diseases
IQR: Interquartile range
MRE: Magnetic resonance enterography
MRI: Magnetic resonance imaging
OR: Odds ratio
PsO: Psoriasis
SI: Sacroiliitis
SIJs: Sacroiliac joints
SpA: Spondyloarthritis
SPAIR: Spectral-attenuated inversion recovery
STIR: Short tau inversion recovery
TNFαi: Tumor necrosis factor α inhibitor
UC: Ulcerative colitis
